# Resting Heart Rate Variability, Perceived Emotion Regulation, and Low-Risk Drug Use in College-Aged Adults: Gender as a Moderator

**DOI:** 10.3389/fpsyt.2022.885217

**Published:** 2022-07-04

**Authors:** Enoch S. Kwon, Ahmad A. Kittaneh, Gina M. Gerardo, Julian Koenig, Julian F. Thayer, DeWayne P. Williams

**Affiliations:** ^1^Department of Psychological Science, University of California, Irvine, Irvine, CA, United States; ^2^Department of Psychology, Kent State University, Kent, OH, United States; ^3^Department of Psychology, The Ohio State University, Columbus, OH, United States; ^4^Department of Child and Adolescent Psychiatry, Psychosomatics and Psychotherapy, Faculty of Medicine and University Hospital Cologne, University of Cologne, Cologne, Germany

**Keywords:** history of drug use, emotion regulation, heart rate variability, motivation, vagal tone, health behaviors, gender, sex differences

## Abstract

Identification of individual differences in drug use is warranted, as a history of use is associated with future drug problems. Such drug use is thought to disrupt inhibitory and motivation networks involved in emotion regulation (ER). Higher resting heart rate variability (HRV), a biomarker of effective inhibitory abilities, is associated with less substance (e.g., alcohol, opioid) use. Higher HRV is associated with lower perceived ER difficulties, and this link is stronger in women relative to men. Evidence suggests women might engage in drug use primarily to reduce stress, and men primarily to induce feelings of elation. Research has yet to examine associations among individuals’ difficulties in ER, resting HRV, and a recent history of drug use; the current study explored this, in addition to how these associations might differ as a function of gender. Young and healthy college students (*N* = 190; 88 women) completed a 5-min baseline to assess resting HRV, followed by the 36-item difficulties in ER Scale and 10-item Drug Abuse Screening Test. Higher difficulties in ER, but not resting HRV, were associated with a greater history of “low-risk” drug use in the full sample and moderation tests confirm this link was stronger in women. Moderated-mediation results confirmed an *indirect* association between resting HRV and drug use, mediated by self-reported difficulties among women only. A significant association between resting HRV and Difficulties in Emotion Regulation Scale (DERS) emerged only among women without a history of drug use. These results indicate that difficulties in ER are both associated with a low-risk history of drug use and underlie an indirect link between resting HRV and drug use history in women only. Among these women with a history of drug use relative to women without, there was no link between resting HRV and self-reported difficulties in ER, suggesting a disrupted inhibitory-motivational pathway. Additional work is needed to understand the psychophysiological correlates of a history of low-risk drug use in young men. These data are in line with research suggesting gender differences in the motivation to engage in recreational drug use and ER interventions might be important in women who engage in low-risk recreational drug use.

## Introduction

The initial decision to engage in recreational drug use is typically voluntary, and with continued use, a person’s ability to exert self-control can become seriously impaired (1, 2). In this regard, the Substance Abuse and Mental Health Services Administration (3) proposes that the earlier people begin to use drugs, the more likely they are to develop an addiction, known as a chronic, relapsing disorder characterized by compulsive drug seeking and use despite adverse consequences (4, 5). Therefore, understanding the biological and motivational correlates of recreational drug use is both necessary and warranted for well-being and longevity.

### Inhibition, Emotion Regulation, and Drug Usage

Drug use elicits powerful emotions that can range from remarkably high states, such as pronounced euphoria, to devastatingly low negative emotional states that in the extreme cause disruption and break with homeostasis (6). Repetitive drug use also produces an abnormal activation of incentive salience/reward systems, such as the release of dopamine and opioid peptides in the extended amygdala, which generally plays a crucial role in guiding behavior toward high-value incentives in the environment (6). Thus, it is clear that neurophysiological pathways underlying emotional-motivational states might be disrupted in individuals who engage in early recreational drug use. Such disruption is indicative of poorer emotion regulation (ER), defined as an individual’s ability to modify their emotional experiences, expressions, and subsequent physiological responses to appropriately respond to ever-changing environmental demands (7). In other words, ER is a mechanism that enables better coping with environmental demands (8, 9). Therefore, it is possible that individuals with more difficulties in ER also have a history of recreational drug use, and vice versa.

Inhibitory control is a necessary component of ER as it involves controlling one’s behaviors and thoughts, potentially overriding a strong internal predisposition or external temptation and choosing the most appropriate or needed response (10, 11). From a neurophysiological perspective, cortical brain regions, such as the prefrontal cortex, exert inhibitory control of subcortical structures, such as the amygdala, thereby allowing the organism to respond to environmental demands adaptively and effectively engaging in self-regulation such as ER (12). Therefore, in a resting state, active cortical brain regions may represent more flexibility in inhibitory control and thus self-regulation (12, 13). Importantly, converging evidence suggested that the reciprocal activity between the neural structures is reflected in autonomic nervous system activity (12). To elucidate the psychophysiological mechanisms connecting inhibition with overall health, Thayer and Lane (13) proposed that characteristic beat-to-beat variability in the heart rate time series – heart rate variability (HRV) – serves not only as an index of healthy heart function (14), but also as a readily available index and measure of inhibitory control, ER ability (11, 15), and overall self-regulatory (e.g., self-control) abilities (12).

### Resting Heart Rate Variability as an Index of Emotion Regulation Abilities

Several neuroimaging and pharmacological studies have identified the link between inhibitory executive brain regions and cardiac parasympathetic activity as indexed by resting HRV (12, 16, 17). The Neurovisceral Integration Model (NIM) postulates that HRV is an index of parasympathetic activity, and thus *resting* HRV serves as a readily available biomarker of self-regulatory (e.g., emotional and cognitive control) abilities. For instance, individuals with higher resting HRV have been shown to exhibit effective behavioral responses (e.g., faster response times and better accuracy) on executive cognitive tasks (18) as well as more flexible and adaptive emotional responding relative to individuals with lower resting HRV (19, 20). In contrast, individuals with the latter pattern exhibit hypoactive prefrontal brain activation, which results in hyperactive subcortical structures that are believed to contribute to maladaptive cognitive and emotional self-regulation (12). Overall, a reciprocal cortico-subcortical inhibitory neural circuit may serve as the structural link between psychological processes such as ER and health-related physiological processes, and this circuit can be indexed by resting HRV (12).

As it relates to substance consequences, lower HRV is associated with greater alcohol problems (21, 22), cravings for alcohol and associated negative mood (23), and non-medical prescription opioid use (24). Furthermore, chronic drug use also tends to be associated with reduced HRV (25). Additionally, previous research showcased the possible feasibility of utilizing an HRV biofeedback intervention (added to a traditional 28-day substance disorder inpatient treatment program) and its efficacy for reducing alcohol and drug cravings (26). Specifically, lower resting HRV was related to increases in craving, whereas higher resting HRV was related to a greater decrease in craving from the start to the end of the treatment (26). This study highlighted the idea that lower resting HRV, marking poorer self-regulation, increases drug use. However, research has yet to link resting HRV with *self-reported* history of drug use. This is warranted as individual differences in resting HRV appear to be a useful index in identifying individuals’ likelihood of engaging in recreational drug use – a gateway to drug problems.

Importantly and in line with the NIM, resting HRV has been linked with self-reported ER difficulties, such that higher resting HRV is linked with lesser perceived difficulties in ER (11, 15, 27). In young and apparently healthy individuals, it has been conceptualized that disruptions in the link between resting HRV and perceived ER difficulties might reflect a lack of consistency between ER capacity and ER motivations, respectively (27). As mentioned, disruptions in neurophysiological pathways underlying emotional-motivational states exist in drug users. Thus, the association between resting HRV and self-reported ER difficulties should be weaker in those with a history of drug use, as those individuals should be less accurate in their ER assessment thereby reflecting lesser disruption in emotional-motivational states. Yet, research has not considered how the association between resting HRV and self-reported ER difficulties might differ between those with and those without a history of drug use.

### Gender Differences

While the gender gap has been decreasing over the past few decades (28), according to the (29) men are more likely than women to use almost all types of illicit drugs, including methamphetamines, cannabis, inhalants, tranquilizers, cocaine, narcotics, and hallucinogens (30). Recent research has found that the propensity for drug use has stayed consistent between genders in that men continued to display riskier behavioral patterns with regard to using illicit substances compared to women (31). Notably, the motivation to initiate drug use differs between men and women. One report proposed men typically engage in drug use to induce feelings of elation, energy, or focus, whereas women might engage in drug use to alleviate high-stress levels, feelings of alienation, depression, anxiety, or post-traumatic stress disorder (28). Additional research also supports a similar gender difference as it relates to the motivation to *initiate* drug use. It has been noted that men primarily misuse prescription opioids to “get high,” whereas women misuse them to help with relaxation and sleep (32), and ER is a relevant factor here. Furthermore, men have been found to typically misuse psychostimulants (e.g., Ritalin, Dexedrine, and Adderall) for reasons related to partying, socializing, increasing sociability, and prolonging the effects of alcohol, while women are more likely to misuse psychostimulants for reasons related to schoolwork, particularly in regards to increased productivity (33). Previous research has also noted that men smoke cigarettes for the reinforcing drug effect of nicotine, whereas women smoke primarily for mood regulation and cue reactivity (34). Taken together, these reports suggest clear gender differences in the motivation to engage in drug use.

Moreover, a meta-analysis suggests that despite having greater heart rate, women also have higher resting HRV compared to men (35). Thus, gender differences in HRV might explain gender differences between men and women in drug use tendencies (i.e., women have greater inhibitory control thus less likely to have a history of drug use). Yet, higher psychopathology (i.e., depression and anxiety) is associated with greater substance issues (36, 37), and thus, ER difficulties may be particularly linked with a history of drug use in women. Relatedly, studies have shown that the negative association between resting HRV and both self-reported ER difficulties (27) and HR (38) is stronger in women than men; these data suggest basic psychophysiological differences between women and men which might extend to drug use tendencies.

Consequently, there is a possibility of gender differences in the relationships among resting HRV, self-reported difficulties in ER, and history of drug use. This is particularly important to consider in a young and apparently healthy population, as it would highlight how a history of drug use in early adulthood is related to psychophysiological processes differentially between men and women. Such results would potentially suggest a differential intervention between genders as it relates to decreasing the likelihood of drug use, supporting Cosgrove et al. (34) suggestion that more gender-sensitive treatments need to be taken into consideration. To this end, research on HRV and substance use has not often considered gender as a factor that may substantially alter such findings.

### Present Study

Emotion regulation is implicated in substance use, including drug use; however, research has yet to consider the association among perceived ER difficulties (i.e., self-reported/subjective ER difficulties), resting HRV (i.e., objective ER abilities), and self-reported history of drug use. Such an investigation would work to understand psychophysiological processes related to drug use. Therefore, our study sought to evaluate the association between a history of drug use and both resting HRV and difficulties in ER. We were particularly interested in these direct associations, in addition to if the link between resting HRV and ER difficulties differed between those with and those without a history of drug use. Finally, we examined whether men and women differed in the above associations.

Considering converging evidence linking substance use with ER processes, we hypothesized a history of drug use to be correlated with lower resting HRV and higher perceptions of ER difficulties. Furthermore, as drug use might disrupt emotional-motivation systems (39), we hypothesized a weaker correlation between resting HRV and ER difficulties in those with relative to those without a history of drug use.

**FIGURE 1:**
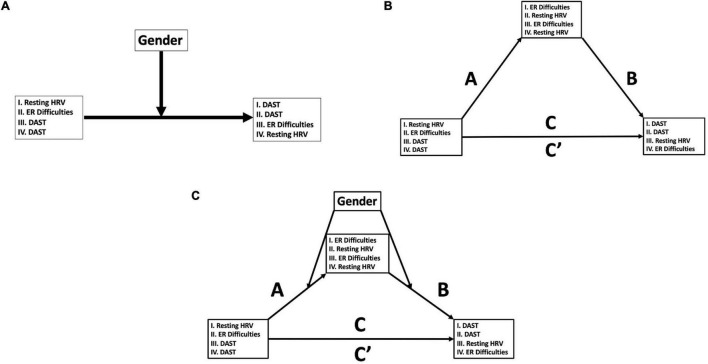
Conceptual moderation, mediation, and moderated-mediation models. **(A)** PROCESS Model 1 (48): moderation models of variables of interest (HRV, heart rate variability; ER, emotion regulation; DAST, Drug Abuse Screening Test). Models 1A and 1B refers to hypothesized models, and Models 1C and 1D are alternative models that were examined (see section “Materials and Methods” for details). **(B)** PROCESS Model 4 (48): mediation models of variables of interest (HRV, heart rate variability; ER, emotion regulation; DAST, Drug Abuse Screening Test). Model 2A refers to the hypothesized model, and Models 2B and 2C are alternative models that were examined (see section “Materials and Methods” for details). **(C)** PROCESS Model 58 (48): moderated-mediation models of variables of interest (HRV, heart rate variability; ER, emotion regulation; DAST, Drug Abuse Screening Test). Model 3A refers to the hypothesized model, and Models 3B and 3C are alternative models that were examined (see section “Materials and Methods” for details).

Given that the link between resting HRV and perceived ER difficulties (11, 15, 27), objective (35) and subjective ER (27), and the rationale for engaging in drug use (28, 32, 33) all differ between genders, it is likely that our hypotheses differ between men and women. We hypothesized that women would show both higher resting HRV and an unlikely history of drug use compared to men. In line with this, if women engage in early drug usage for feelings of stress in contrast to men who may engage in drug usage for feelings of elation, both resting HRV and self-reported ER difficulties should be more strongly associated with drug usage in women compared to men. In other words, we hypothesized that gender would moderate or alter the association between ER processes and a history of drug use (see Figures 1A-I,A-II for hypothesized conceptual model). Finally, the negative association between resting HRV and ER difficulties appears stronger in women (27), therefore we hypothesized that the correlation between resting HRV and ER difficulties should be particularly weaker in women with a history of drug use relative to women without.

As an exploratory analysis, we examined the potential mediated (i.e., three-way or indirect) association between resting HRV, ER difficulties, and a history of drug use (see Figure 1B for possible conceptual models) and if gender moderated this model (see Figure 1C for possible conceptual models).

## Materials and Methods

### Participants

Archival data from two pooled studies conducted within the Emotions and Quantitative Psychophysiology Lab at The Ohio State University were combined for the current study. The primary focus of one study was to consider ethnic differences in the psychophysiological correlates of pain [see (40) for additional methods and procedure details], and the other study focused on false memory [see (41) for similar methods and procedure]. In both studies, participants were recruited *via* two methods: (1) a Research Experience Program (REP) pool at The Ohio State University, which allowed students to participate in research for partial class credit in an introductory level psychology course, and (2) cash compensation for individuals’ participation outside of the research pool. A total of 190 participants (102 males, 88 females; 88 ethnic minorities; *M*_age_ = 20.07, SD = 2.87, age range: 18–38 years) were available for analysis. Of the 190 participants, 63 of the present study participants were included in Williams et al. (27). Participants younger than 18 years old and/or those who were allergic to adhesives were not able to participate in either study.

### Procedure

In both studies, participants were asked not to smoke, undergo vigorous physical activity, or drink caffeine 6 h prior to the start of the experimental session. Each study was approved by the Institutional Review Board (IRB) at The Ohio State University, and all participants signed written informed consent. In both studies, participants were placed in a soundproof experimental room equipped with a camera and microphone for safety and instructional reasons and a high-definition TV for stimuli presentation. Participants were given a detailed explanation of the procedures that would occur without indicating the specific hypothesis under the study or manipulations applied. Electrocardiogram leads were attached to the subjects, and while in a separate control room, the experimenter led the subjects through the initial phases of the experiment. All participants first completed a 5-min baseline-resting period, which included viewing a blank, gray screen. They were told not to move or fall asleep and to simply relax and breathe normally. Participants then completed a set of self-report questionnaires; importantly, the questionnaires were administered prior to any experimental procedure in both studies.

### Resting Heart Rate Variability

Cardiac activity data were recorded continuously throughout each experiment *via* a three-lead ECG at a 1000 Hz sampling rate using a Mindware™ 2000D (MW2000D) Impedance Cardiograph package. Resting vmHRV was assessed during a 5-min baseline (spontaneous breathing and resting state) period prior to any experimental task. Electrodes were placed (1) below the right clavicle, (2) on the left side of the abdomen (below the heart), and (3) on the right side of the abdomen. The variability between successive R-spikes (or variability within inter-beat-intervals, IBIs) was obtained from ECG recordings to calculate HRV. Participants’ successive IBIs, in milliseconds, were extracted using HRV 2.51 Analysis software. IBIs were written in a text file and analyzed using Kubios HRV analysis package 2.0 (42), allowing for the calculation of time-and frequency-domain indices of HRV. Artifacts within the R-to-R series were visually detected. An artifact correction level that would differentiate and remove artifacts (differing abnormal IBIs from the mean IBI) using a piecewise cubic spline interpolation method was employed. The root mean square of successive differences (RMSSD), measured in milliseconds, was calculated and is considered to be a stable (43) and valid (44, 45) time-domain measure of HRV. Autoregressive estimates were also calculated, yielding high-frequency power HRV (HF-HRV, 0.15–0.4 Hz) (44, 45). In the present study, RMSSD correlated highly with HF power (*r* = 0.90, *p* < 0.001). For ease of interpretation, only HRV results using HF-HRV are reported, although results were virtually identical using RMSSD. HF-HRV values were natural log-transformed (ln) to fit assumptions of linear analyses (45).

### Self-Report Questionnaires

Perceived difficulties in ER were assessed *via* self-report using the Difficulties in Emotion Regulation Scale (DERS; completed within 30 min of the baseline-resting period described above). The DERS is comprised of 36-items and 6 sub-scales designed to measure different facets of difficulties in ER (46). Participants are asked to respond on a scale from 1 (*almost never*) to 5 (*almost always*) regarding how much these statements are reflective of them (example item: “*When I’m upset, I believe that I will end up feeling very depressed*”). Subscales included (a) *difficulties in controlling impulsive behavior when experiencing negative emotions* (impulse); (b) *lack of strategies to regulate emotions* (strategies); (c) *lack of emotional awareness* (awareness); (d) *non-acceptance of emotional responses* (non-accept); (e) *lack of emotional clarity* (clarity); and (f) *difficulties engaging in goal-oriented behavior when experiencing negative emotions* (goals). The DERS total score is based on all 36-items, and subscales were calculated in accordance with prior psychometric studies.

Drug use history (over the prior 12 months) was assessed using the Short Form Drug Abuse Screening Test (DAST-10), a 10-item self-report scale adapted from the original 28-item DAST (47). Participants answer YES or NO on each of the 10 questions. A score of “1” is given for each YES response, and a score of “0” is given for each NO response. Higher scores are indicative of a higher risk of drug use. According to this scale, score labels are as follows: a score of zero – No problems reported; a score of 1–2 – Low level; a score of 3–5 – Harmful; a score of 6–8 – Substantial level; and a score of 9–10 – Severe level. All participants (*n* = 190) included in this study scored between 0 and 2, and thus, all individuals fell between “no risk” or those without a history of drug use, and “low-risk” or those with a history of drug use.

### Statistical Analyses

Participants were stratified into groups based on their self-reported gender. In addition to keeping DAST scores as a “continuous” variable (scores 0–2), participants were also divided into two groups based on their DAST scores; those who scored a total score of zero were considered to be the “no risk” or no history of drug use group, and those who scored one or two points were grouped as the “low-risk” or history of drug use group.

Correlations were used to determine associations between variables of interest. Due to the non-normal distribution of DAST scores, all correlation coefficients between DAST scores and other variables are represented as Spearman’s rank correlation coefficients (ρ), while all other correlation coefficients represent Pearson’s *r*. These correlations were conducted in the entire sample as well as stratified by gender.

To test if gender moderated the relationship between resting HRV and DERS scores on DAST scores, the SPSS-macro PROCESS was used (48). In PROCESS, “Model 1” was used to test the moderating effect of the independent variables (IV; HF-HRV, DERS), a conditional effect of the moderator (M; gender), and an interaction effect of the two on the dependent variable (DV; DAST scores) (see Figures 1A-I for conceptual representation for HRV model; see Figures 1A-II for conceptual representation for DERS model). Conditional effects (48) were used to probe potential differential associations of the moderator (i.e., gender). In this regard, high and low values for the predictor variables are derived using ±1 SD from the mean, allowing the PROCESS program to yield predicted DV values at varying levels of the predictor variable *via* regions of significance and simple slope analyses. Models using DAST scores as IVs and both DERS (see Figures 1A-III for conceptual representation) and resting HRV (see Figures 1A-IV for conceptual representation) as DVs were also tested as alternatives.

In PROCESS (48), Model “4” was also used to explore potential mediation effects between resting HRV, DERS, and DAST scores. In this test, resting HRV was the IV with the mediating variable as DERS scores, with and DAST scores as the DV (see Figures 1B-I for conceptual representation). Alternative models were also considered, with DAST scores as the IV, DERS scores as the mediating variable, and resting HRV as the DV (see Figures 1B-III for conceptual representation), in addition DAST as the IV, M as resting HRV, and DERS scores as the DV (see Figures 1B-IV for conceptual representation). Finally, gender was considered the moderating variable in “Model 58” in PROCESS (48), which represents moderated mediation. That is, we tested if gender moderates a possible mediation model proposed among HRV, DERS, and DAST scores (see Figure 1C for all tested conceptual models). For both Models 4 and 58, statistics for Paths A (IV-M), B (M-DV), C (direct IV-DV), and C’ (indirect IV-DV) are reported. Statistics include unstandardized beta (B) coefficients, standard errors (SEs; in brackets), 95% bootstrapping confidence intervals [95% boot CI in square brackets, 5000 samples; (48)], partial correlation coefficients (for main effects and interactions), and *p*-values.

In all PROCESS (48) analyses, several covariates were considered. Ethnicity has a non-trivial association with resting HRV (49, 50) and thus, was included as a covariate in applicable analyses (ethnicity coded as 1 = White, 2 = Black, 3 = Asian, 4 = Hispanic, 5 = Middle Eastern, 6 = Other). Higher body mass index (BMI) is also associated with decreased resting HRV [e.g., (51, 52)]; in the current sample, men showed greater BMI compared to with women (see section “Results” for details) and thus, BMI was also used as a covariate in applicable analyses. Gender was also included as a covariate for PROCESS models that did not include gender as a predictor variable.

All statistical tests were conducted using SPSS (ver. 27, IBM, Chicago, IL, United States). All tests were two-tailed, and significance levels were evaluated using an alpha of 0.05.

## Results

### Participant Demographics

Means and standard deviations for all variables of interest, in addition to gender differences, are presented in Table 1. Women had marginally higher DERS (*M* = 84.47, SD = 20.15); [*t*(188) = −1.716, *p* = 0.044, η = 0.124] and strategies subscale (*M* = 16.32, SD = 6.29); [*t*(188) = −1.87, *p* = 0.032, η = 0.135] scores compared to men (DERS: *M* = 80.00, SD = 15.69; *M* = 14.83, SD = 4.64). There were no significant differences between men and women on resting HRV [*t*(188) = 0.50, *p* = 0.309, η = 0.037] or DAST scores; [*t*(188) = −0.47, *p* = 0.318, η = 0.035].

**TABLE 1:** Mean differences between men and women on variables of interest.

	Total	Men	Women	*F*	*r*	*p*
*N*	190	102	88			
BMI	24.78 (6.13)	25.14 (4.96)	24.36 (7.27)	0.77	0.063	0.383
Mean HR	75.50 (11.45)	75.04 (11.79)	76.03 (11.45)	0.35	0.044	0.554
Resting HRV	6.74 (1.01)	6.720 (1.03)	6.760 (0.97)	0.09	0	0.764
Drug use	0.46 (0.69)	0.440 (0.70)	0.490 (0.68)	0.22	0.032	0.636
Total DERS	82.07 (17.98)	80.00 (15.69)	84.47 (20.15)	2.94	0.122	0.088
*Impulse*	11.03 (4.02)	10.75 (3.14)	11.35 (4.85)	1.04	0.077	0.308
*Strategies*	15.52 (5.50)	14.83 (4.64)	16.32 (6.29)	3.48	0.134	0.064
*Awareness*	18.85 (4.02)	18.98 (4.83)	18.70 (4.84)	0.15	0.032	0.695
*Non-accept*	11.58 (5.39)	10.80 (4.86)	12.48 (5.84)	4.65	0.155	0.032*
*Clarity*	11.99 (3.09)	11.70 (2.96)	12.34 (3.23)	2.06	0.104	0.152
*Goals*	13.09 (3.69)	12.93 (3.33)	13.27 (4.09)	0.4	0.044	0.527

This table presents the means and standard deviations (in brackets) on variables of interest both in the full sample and stratified by women and men. MANOVA statistics include F- and p-values along with effect size represented by eta (r). Significant differences bolded. HRV, heart rate variability; HR, heart rate; DERS, difficulties in emotion regulation (subscales italicized); BMI, body mass index. *p < 0.05.

### Zero-Order Correlations Among Variables of Interest

Correlations among the variables of interest in the entire sample are presented in Table 2A. In the full sample, higher DAST scores were related to higher DERS (ρ = 0.196, *p* = 0.007) and impulse subscale (ρ = 0.202, *p* = 0.005) scores. Resting HRV was not significantly correlated with any variables of interest at a bivariate level. Stratified by men and women (Table 2B), resting HRV was negatively correlated with DERS (*r* = −0.238, *p* = 0.026) and DERS-strategies subscale scores (*r* = −0.288, *p* = 0.007) for women. Drug use scores were positively correlated with DERS (ρ = 0.336, *p* = 0.001), DERS-impulse subscale (ρ = 0.346, *p* < 0.001), and DERS-strategies subscale (ρ = 0.222, *p* = 0.037) scores for women. No significant or notable associations were found among men. Stratified by drug group (Table 2C), resting HRV was negatively correlated with BMI (*r* = −0.217, *p* = 0.016) for the no-risk group. There were no significant or notable associations amongst the low-risk group.

**TABLE 2:** Correlation coefficients for full sample and stratified by gender and drug group.

A		1	2	3	4	5	6	7	8	9	10
1	Drug use	–									
2	Resting HRV	−0.019	–								
3	Total DERS	0.196**	−0.105	–							
4	*Impulse*	0.202**	−0.080	0.822**	–						
5	*Strategies*	0.126	−0.128	0.870**	0.729**	–					
6	*Awareness*	0.021	−0.069	0.292**	0.091	−0.015	–				
7	*Non-accept*	0.084	−0.133	0.746**	0.549**	0.663**	−0.067	–			
8	*Clarity*	0.107	0.054	0.631**	0.490**	0.445**	0.313**	0.215**	–		
9	*Goals*	0.202**	0.004	0.680**	0.497**	0.630**	−0.123	0.496**	0.317**	–	
10	BMI	0.034	−0.147*	0.104	0.121	0.084	0.080	0.050	0.101	−0.010	–

**B**		**1**	**2**	**3**	**4**	**5**	**6**	**7**	**8**	**9**	**10**

1	Drug use	–	−0.071	0.336**	0.346**	0.222*	0.052	0.174	0.158	0.408**	−0.065
2	Resting HRV	0.023	–	−0.238*	−0.140	−0.288**	0.020	−0.305**	0.017	−0.168	−0.274**
3	Total DERS	0.052	0.022	–	0.853**	0.898**	0.172	0.817**	0.656**	0.648**	0.118
4	*Impulse*	0.067	−0.015	0.776**	–	0.767**	−0.017	0.654**	0.567**	0.479**	0.072
5	*Strategies*	0.030	0.033	0.822**	0.661**	–	−0.116	0.759**	0.531**	0.615**	0.183
6	*Awareness*	−0.006	−0.140	0.443**	0.245*	0.112	–	−0.060	0.223*	−0.227*	0.043
7	*Non-accept*	−0.013	0.021	0.643**	0.385**	0.515**	−0.069	–	0.279**	0.505**	0.147
8	*Clarity*	0.064	0.082	0.596**	0.385**	0.324**	0.407**	0.118	–	0.292**	0.060
9	*Goals*	−0.007	0.171	0.723**	0.531**	0.655**	−0.011	0.484**	0.341**	–	−0.095
10	BMI	0.145	−0.002	0.108	0.232*	−0.058	0.126	−0.070	0.179	0.129	–

**C**		**1**	**2**	**3**	**4**	**5**	**6**	**7**	**8**	**9**	

1	Resting HRV	–	−0.112	−0.100	−0.114	−0.076	−0.161	0.136	−0.023	−0.034	
2	Total DERS	−0.104	–	0.866**	0.917**	0.088	0.734**	0.576**	0.704**	−0.026	
3	*Impulse*	−0.065	0.780**	–	0.777**	−0.025	0.581**	0.500**	0.534**	0.058	
4	*Strategies*	−0.143	0.830**	0.667**	–	−0.101	0.644**	0.487**	0.704**	−0.041	
5	*Awareness*	−0.064	0.422**	0.177	0.040	–	−0.227	−0.066	−0.240	0.091	
6	*Non-accept*	−0.114	0.758**	0.531**	0.681**	0.023	–	0.255*	0.471**	−0.218	
7	*Clarity*	−0.002	0.656**	0.469**	0.399**	0.534**	0.181*	–	0.311*	0.052	
8	*Goals*	0.023	0.641**	0.425**	0.551**	−0.060	0.510**	0.294**	–	0.027	
9	BMI	−0.217*	0.175	0.164	0.163	0.073	0.193*	0.124	−0.044	–	

Table A represents correlations between variables of interest for the full sample (n = 190). Table B represents correlations between variables of interest stratified by gender with men on the left side of the diagonal and women on the right. Finally, Table C represents correlations between variables of interest stratified by drug group with no risk individuals on the left of the diagonal and low risk individuals on the right. Significant correlations bolded. HRV, heart rate variability; DERS, difficulties in emotion regulation (subscales italicized); BMI, body mass index. *p < 0.05, **p < 0.01.

**FIGURE 2:**
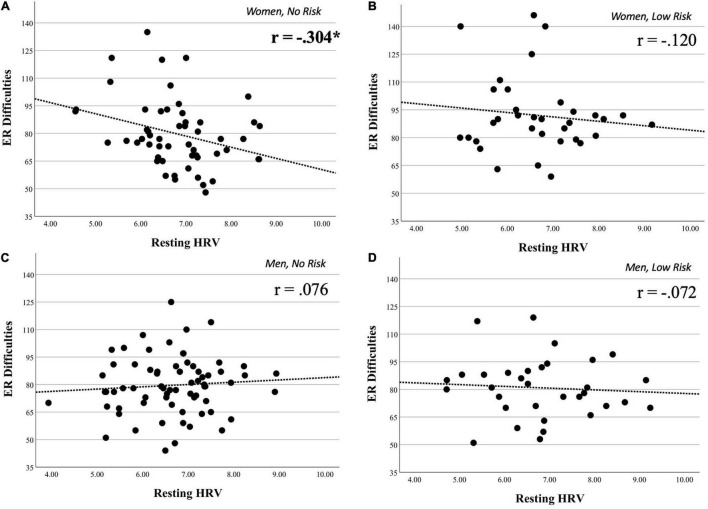
Scatterplots on the association between resting HRV and ER difficulties on groups of interest. Scatterplots illustrating the associations between resting heart rate variability (HRV) and Difficulties in Emotion Regulation Scale (DERS) scores on four major groups of interest. These groups were obtained through the stratification of our full sample by gender and drug group. Panel **(A)**, which represents women **with no history of drug use**, shows the only significant association between HRV and DERS. Panel **(B)** (**women with a history of drug use**), panel **(C)** (**men with no history of drug use**), and panel **(D)** (**men with a history of drug use**) all show non-significant associations between HRV and DERS (see section “Materials and Methods” for details). *p < 0.05.

The association between resting HRV and DERS was significant in no-risk women (*r* = −0.304, *p* = 0.025), but not in low-risk women (*r* = −0.120, *p* = 0.500), no-risk men (*r* = 0.076, *p* = 0.533), or low-risk men (*r* = −0.072, *p* = 0.692). See Figure 2 for scatterplots of these associations by group; results remain the same considering covariates.

### Moderation, Mediation, and Moderated-Mediation Analyses

**FIGURE 3:**
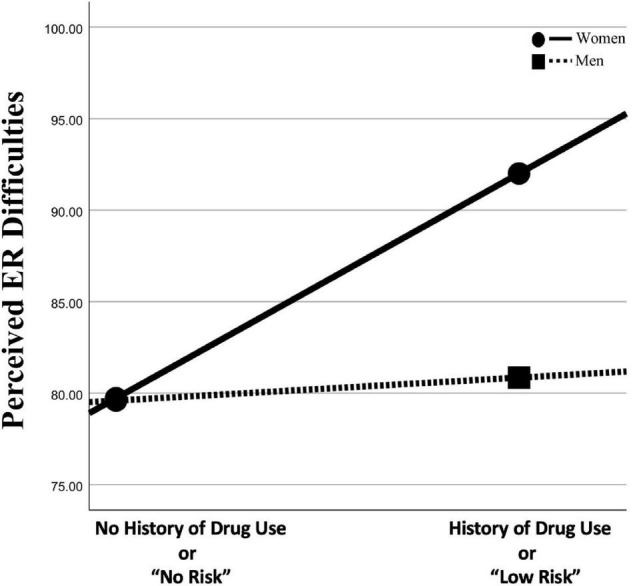
Conditional effects of moderation test. This figure depicts the association between drug use and emotion regulation (ER) difficulties moderated by gender (see section “Materials and Methods” for details).

Moderation analyses showed that gender significantly moderated the association between DAST and DERS scores [*B* = 12.67 (5.35), 95% boot CI [2.11, 23.24], *r*_partial_ = 0.30, *p* < 0.05]. Conditional analyses showed that women [*B* = 12.74 (3.82), 95% boot CI [5.21, 20.27], *p* < 0.01] compared with men [*B* = 0.07 (3.72), 95% boot CI [−7.28, 7.41], *p* = 0.99] showed a stronger association between DAST and DERS scores (see Figure 3). A similar moderation effect of gender was also found on the association between DAST and the DERS-impulse subscale [*B* = 2.75 (1.2), 95% boot CI [0.39, 5.11], *r*_partial_ = 0.30, *p* < 0.05]. Conditional analyses showed that women [*B* = 3.05 (0.85), 95% boot CI [1.36, 4.73], *p* < 0.001] compared with men [*B* = 0.29 (0.83), 95% boot CI [−1.35, 1.94], *p* = 0.72] showed a stronger association between DAST and DERS-impulse (not graphically represented).

Gender did not significantly moderate the association between drug use and HRV [*B* = −0.15 (0.22), 95% boot CI [−0.57, 0.28], *p* = 0.493]. Similarly, moderation analyses showed that gender did not significantly moderate the association between drug use and difficulties in ER [*B* = 6.463 (3.74), 95% boot CI [−0.92, 13.84], *p* = 0.086].

**FIGURE 4:**
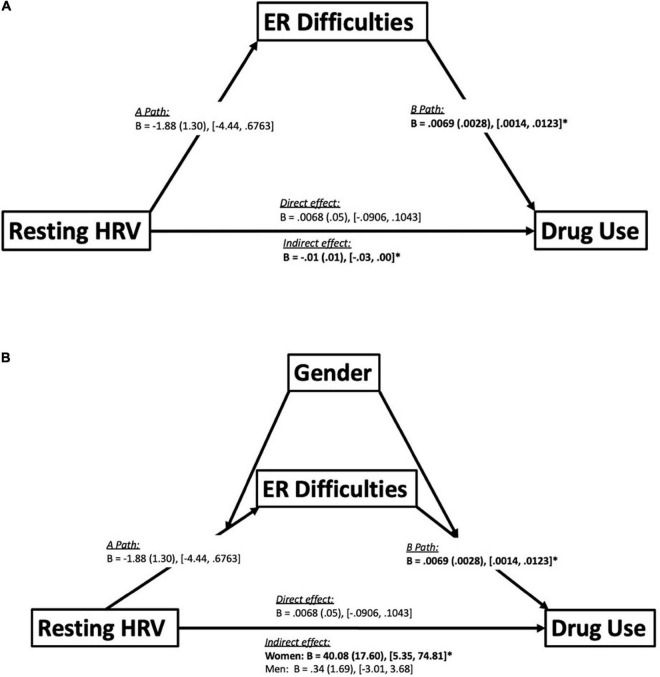
Mediation and moderated-mediation model. Panel **(A)** depicts a mediation model illustrating emotion regulation (ER) difficulties mediating an indirect association between resting HRV and drug use. Statistics reported include unstandardized betas (B), standard error (in brackets) and the bootstrapping CI’s (lower limit, upper limit) for each path of the model. Path A represents the association between the independent variable (resting HRV) and ER difficulties. Path B represents the association between ER difficulties and drug use. Furthermore, there were also direct and indirect paths between resting HRV and drug use. The direct path represents the direct effect between resting HRV and drug use. The indirect path represents the indirect effect of resting HRV on drug use through ER difficulties. Significant effects bolded. *p < 0.05 (see section “Materials and Methods” for details). Panel **(B)** depicts a moderated-mediation model illustrating the effects of utilizing gender as a moderator on the mediation analysis seen in **(A)**. Statistics reported include unstandardized betas (B), standard error (in brackets) and the bootstrapping CI’s (lower limit, upper limit) for each path of the model. The moderation of gender revealed that the indirect effect between resting heart rate variability (HRV) and drug use mediated by difficulties in emotion regulation (ER), was only significant amongst women but not men. Significant effects bolded. *p < 0.05 (see section “Materials and Methods” for details).

In the full sample, DERS mediated an *indirect* association between resting HRV and DAST scores [*B* = 0.007 (0.003), 95% boot CI [0.0014, 0.0123], *p* < 0.05], such that lower resting HRV was associated with higher DERS scores, but not statistically significant [Path A: *B* = −1.88 (1.30), 95% boot CI [−4.44, 0.68], *p* = 0.149]. Higher DERS scores were associated with higher DAST scores [Path B: *B* = 0.007 (0.003), 95% boot CI [0.001, 0.012], *p* < 0.05], and the indirect effect was significant [*B* = −0.01 (0.01), 95% boot CI [−0.03, 0.00]] (see Figure 4A for graphical representation). It is important to note that, and as expected given the correlation analyses, the direct effect was not significant [*B* = 0.007 (0.05), 95% boot CI [−0.091, 0.104], *p* = 0.89]. Gender also moderated this mediation analysis [*B* = 40.08 (17.60), 95% boot CI [5.35, 74.81], *p* < 0.05] such that this indirect effect was present only in women [*B* = −4.93 (1.95), 95% boot CI [−8.78, −1.09], *p* < 0.05] but not men [*B* = 0.34 (1.69), 95% boot CI [−3.01, 3.68], *p* = 0.84] (see Figure 4B).

In the full sample, DERS did not significantly mediate a link between DAST scores and resting HRV [*B* = −0.006 (0.004), 95% boot CI [−0.014, 0.002], *p* = 0.149; see Figures 1B-III for conceptual model]. It is also important to note that the direct effect was not significant [*B* = 0.015 (0.108), 95% boot CI [−0.199, 0.229], *p* = 0.890]. Gender also did not moderate this mediation analysis [*B* = 1.228 (3.097), 95% boot CI [−4.883, 7.338], *p* = 0.692; see Figures 1C-III for conceptual model].

Furthermore, in the full sample, resting HRV did not significantly mediate the association between DAST scores and DERS [*B* = −1.852 (1.279), 95% boot CI [−4.374, 0.670], *p* = 0.149; see Figures 1B-IV for conceptual model], however, the direct effect was significant [*B* = 4.670 1.872, 95% boot CI [0.977, 8.364], *p* = 0.014]. Gender did not moderate this mediation analysis [*B* = 0.114 (0.179), 95% boot CI [−0.238, 0.466], *p* = 0.524; see Figures 1C-IV for conceptual model].

## Discussion

The primary goals of the present study were: (1) to examine the association between a history of drug use and both resting HRV and ER difficulties; (2) to explore if a history of drug use impacts the link between resting HRV and difficulties in ER; and (3) investigate gender differences in these associations.

Correlation and moderation tests partially supported our hypotheses in that there was a direct association between a history of drug use and ER difficulties, and this link was primarily evident in women. No link between resting HRV and drug use was found in men or women. Furthermore, showing a stronger link between resting HRV and difficulties in ER in women compared to men as in Williams et al. (27); importantly, novel data suggested no link between resting HRV and self-reported ER difficulties in women with a history of drug use. This finding potentially characterizes a disruption between ER capacities, marked by resting HRV, and ER difficulties, marked by self-reported ER difficulties, in women with a low-risk history of drug use. Among the six different facets of ER difficulties, only those related to difficulties with impulse control and ER strategies were most related to low-risk drug use in women but not men. Finally, while correlational analyses confirm resting HRV was not *directly* associated with DAST in the full sample or stratified by gender, our explorative mediation results suggested an *indirect* association between resting HRV and drug use mediated by self-reported difficulties in ER. However, this mediated association was also moderated by gender, such that this model was statistically reliable in women but not men.

Overall, this is the first study to explore how both resting HRV and difficulties in ER are related to drug usage and how these associations may differ as a function of gender. Women initiate drug use primarily for stress regulation (28), and our data are in line with this idea, as in women only, perceived ER difficulties were associated with drug use, self-reported difficulties in ER mediated the resting HRV and drug use link, and a history of drug use was associated with a weaker link between resting HRV and self-reported ER difficulties. Substantial research remains needed to understand appropriate mechanisms to decrease the likelihood of drug use in men, especially from an ER standpoint. Indeed, men tend to underreport symptomology, and thus, it is possible that with more data, the associations within men might be highlighted. As mentioned, research suggests men initiate drug use for the thrill, drive, and fun of the experience, and our study was not able to determine such motivations. Therefore, and alternatively, other self-regulatory scales need to be considered, such as impulsiveness and drive-seeking behaviors, to understand the biological-motivational factors that drive drug behavior in men. In sum, these findings suggest that differences between men and women exist as it relates to the potential ER-related catalyst of drug use; perceived ER difficulties may drive low-risk drug behavior for women more than men, and that resting HRV and ER difficulties are only linked in low-risk women. These findings are in line with prior studies showing a disruption in the inhibitory pathway in those who engage in heavy alcohol use (53).

Another finding that was unexpected, yet unsurprising, was that among those without a history of drug use (both men and women), higher resting HRV was associated with lower BMI. Obesity is considered an inflammatory process (54), and resting HRV represents the physiological pathway underlying inflammatory processes (55). Thus, higher vagal activity, as indexed by higher resting HRV, should be associated with lower measures of adiposity (e.g., BMI) *via* proper regulation of the anti-inflammatory cholinergic pathway. As such, the link between resting HRV and BMI among those without a history of drug use appears intuitive and further supports the idea of a disrupted inhibitory pathway among those with a history of drug use. However, and importantly, this finding should be interpreted with extreme caution, especially given that our sample fell within the normal BMI range and no-to-low risk of drug use. Therefore, future research is needed to directly address how and if a history of drug use impacts the associations between resting HRV and indices of adiposity (i.e., BMI).

Overall, our data suggest that even for those considered low-risk, ER difficulties and resting HRV (indirectly) can be used to predict a history of drug use which potentially serves as a gateway to drug problems. Importantly, a lack of association between resting HRV and ER difficulties may signal a history and/or likelihood of drug use in women, and vice versa.

### Implications

Our data suggest that a history of drug use, even at a low risk, is associated with a weaker link between resting HRV and ER difficulties among women. An alternative interpretation is that being accurate in one’s assessment of ER capacities and subsequent ER difficulties may be key in decreasing the likelihood of young women to ever engage in low-risk drug use. It is interesting to consider that the stronger association between resting HRV and ER difficulties in women relative to men (27) might be a contributing factor underlying lesser drug use in women relative to men (although not supported in the current study). Thus, given that this association appears important in low-risk women in our sample, it is likely very impactful in those struggling with addiction more generally, and future research should investigate this possibility directly. Regarding men, it is not surprising the link between resting HRV and ER difficulties did not reach statistical significance as a similar effect size was found for men in our prior report (11).

Furthermore, and from a clinical standpoint, the target of decreasing the likelihood of recreational drug use in women is likely ER-based, whereas for men, it may be based on motivational factors unrelated to ER (i.e., thrill-seeking) and more research is needed in this regard. Given the indirect association of resting HRV and drug use among women, one promising intervention may be HRV biofeedback, which has been shown to reduce cravings among inpatient young men (26). In women, this feedback might be promising in that it may strengthen the understanding of one’s own ER capabilities. An accurate assessment here allows the individuals to be more motivated in ER processes (i.e., lesser perceived ER difficulties), or on the other hand, motivated to change the capacity *via* additional HRV biofeedback sessions, which over time, may increase motivation to engage in ER and thus avoid drug use. Future studies are needed to explore these possibilities.

The present data also suggest that a history of drug use might impair an important psychophysiological compensatory mechanism in young adult women, marked by a disrupted link between resting HRV and ER (27). On the other hand, men appear to engage in drug use irrespective of ER processes which is particularly problematic from clinical, psychotherapeutic, and behavioral change perspective. Research is thus needed to understand psychological and physiological predictors and correlates of drug use in young adult men. Overall, perceived ER difficulties, as a primary factor, and resting HRV, potentially as a secondary factor – should be considered when targeting behavior change related to drug use, especially in young adult women.

### Limitations and Future Directions

One limitation of the current study is that it was cross-sectional by nature and causation cannot be determined. It is certainly plausible that due to the nature of our scale (drug use over the past 12 months), history of drug use could impact resting HRV and ER difficulties, as opposed to resting HRV and ER predicting a history of drug use. Statistically, our results support the latter, as models involving history of drug use as an independent variable and resting HRV and ER difficulties as DVs were not significant. These data suggest ER processes as the predictor of drug use history. Likewise, in mediation models, ER difficulties did not predict resting HRV. From a theoretical standpoint, this makes sense as resting HRV is considered an endophenotype (56) and thus more stable over time relative to both perceived ER difficulties and recreational drug use (especially between no-risk and low-risk levels). Nonetheless, we propose, and prior research suggests (6, 21), substance use is detrimental for psychophysiological function. Therefore, it is important that future studies track changes in HRV, ER difficulties, and drug use over time to understand causal (and likely bidirectional) links.

Relatedly, another limitation is that all participants fell between the “no-risk” or “low-risk” category based on the Short Form DAST-10 (i.e., individuals who scored less than 3 on the DAST). A more direct effect of resting HRV on DAST may exist with a larger and more diverse sample. Future studies should also examine how the link between resting HRV and ER difficulties might diminish *over time* in those who engage in recreational drug use. It will be imperative for future work to understand how interventions might impact both difficulties in ER and resting HRV over time to avoid initial drug use and/or relapse into a drug use disorder. Future research should also work to examine the association between resting HRV and drug use in individuals of different age and ethnic groups.

Another limitation of the study is that we did not assess the duration, frequency, recency, or reasons/motivations of recreational drug use, and therefore it is difficult to determine how these play a role in the current results. Future studies should replicate this work and be understood within the context of these variables to gain a clearer picture of these associations. Finally, we did not assess other substance use, such as alcohol, which is a limitation considering our college sample. High-risk drinking that may be present in this sample may contribute directly to the history of drug use and the disruption between psychophysiological measure. Therefore, future studies must explore these results in the context of other substances, in addition to the frequency and duration of such substances.

However, our study is not without strengths; this is the first study to show associations between ER processes and a history of drug use even in those considered low-risk. Second, this is one of the few studies to consider gender differences in psychophysiological data, especially as it relates to drug use. Finally, we utilized mediation and moderation analyses as a means to understand how these variables are intercorrelated, which we hope will provide fruitful avenues for future research.

## Conclusion

This was the first study, in a young and apparently healthy population within a non-pathological range of drug use scores, to link perceived ER difficulties with a history of low-risk drug use. Higher ER difficulties are associated with a history of drug use, and such a history of drug use disrupts the link between resting HRV and ER difficulties, particularly among young adult women. These findings are in line with studies suggesting women engage in drug use for stress regulation (28, 32, 33). Moreover, more perceived ER difficulties carried the indirect association between lower resting HRV and low-risk drug use history in women. In conclusion, lesser perceived ER difficulties, higher resting HRV, and a stronger link between the two is particularly important in decreasing the likelihood of recreational drug use in college-aged women relative to college-aged men.

## Data Availability Statement

The raw data supporting the conclusions of this article will be made available by the authors, without undue reservation.

## Ethics Statement

The studies involving human participants were reviewed and approved by The Ohio State University Institutional Review Board. The patients/participants provided their written informed consent to participate in this study.

## Author Contributions

EK wrote the initial draft of the manuscript. DW contributed to writing some elements of the manuscript. EK and DW performed the statistical analyses. AK, GG, and JK collected and processed the data. JT, JK, GG, and DW oversaw the study. All authors contributed to the theorizing and conceptualization of the study. All authors contributed to the revision, read, and approval of the final manuscript.

## Conflict of Interest

The authors declare that the research was conducted in the absence of any commercial or financial relationships that could be construed as a potential conflict of interest.

## Publisher’s Note

All claims expressed in this article are solely those of the authors and do not necessarily represent those of their affiliated organizations, or those of the publisher, the editors and the reviewers. Any product that may be evaluated in this article, or claim that may be made by its manufacturer, is not guaranteed or endorsed by the publisher.
